# Study on regulators of purifying magnesite ore by cationic reverse flotation

**DOI:** 10.1038/s41598-021-96549-4

**Published:** 2021-08-20

**Authors:** Pengcheng Li, Shujuan Dai, Wenhan Sun, Mingyang Fan

**Affiliations:** 1 School of Chemical Engineering, University of Science and Technology Liaoning, Anshan, 114051 China; 2 School of Resources and Civil Engineering, Northeastern University, Shenyang, 110819 China

**Keywords:** Mineralogy, Engineering

## Abstract

The floatability of magnesite, dolomite and quartz is a major factor affecting the removal of silicon and calcium from magnesite ore. The effect of the regulators sodium hexametaphosphate (SH), sodium silicate (SS), trisodium phosphate (TP), tannic acid (TN) and monoammonium oxalate (OA) on the floatability of magnesite, dolomite, and quartz under the ether amine (EAH) system was studied. The results show that the five regulators have relatively little influence on the floatability of quartz and magnesite. Dolomite can be activated when the dosage of SH is less than 40 mg/L; however, dolomite can be inhibited when the SH dosage is greater than 40 mg/L. The other four regulators have inhibitory effects on dolomite, and TN and TP have strong inhibitory effects on dolomite. Under the conditions of optimum grinding fineness, pH and collector dosage, a recovery of approximately 70% and a concentrate with a grade of over 47% were obtained by three stages of reverse flotation using sodium hexametaphosphate and water glass as regulators and Haicheng magnesite ore with an SiO_2_ content of 2.38% and a CaO content of 0.75%. Potentiometric measurements and infrared spectroscopy analysis show that physical adsorption occurs between the three minerals and collectors, while the interaction of magnesite and dolomite with SH and SS involves both physical adsorption and chemical adsorption.

## Introduction

Magnesite is a dominant mineral resource in China, which not only ranks first in the world in reserves but also accounts for more than 60% of the world's output due to the good quality of this mineral. China's magnesite is mainly used for refractory materials, which account for more than 80% of its mining volume. At the same time, the metallurgical industry consumes the most magnesium refractories, accounting for 70% of the total refractory materials^1–3^. With the development and utilization of magnesite ore resources, the number of high-quality resources is decreasing, and the existing stockpiled or unmined magnesite ore cannot meet the quality requirements for refractory materials. Therefore, impurity removal and purification of low-grade magnesite ore becomes extremely critical. The main useful mineral in magnesite ore is magnesite, and the gangue minerals are mainly talc, quartz and dolomite. Flotation is the most commonly used and most effective method for magnesite beneficiation and purification, and research on flotation reagents is more important. The main impurities in magnesite are silicates and carbonates. In view of the properties of magnesite, more research and development on collectors and modifiers have been conducted, and an appropriate amount of modifiers can be combined with collectors to more effectively separate magnesite from the gangue minerals^3–9^.

To improve the separation efficiency of magnesite and quartz in reverse flotation, Wang Jinliang^10^ and others developed a new regulator KD-1 and studied its effect in the flotation test of pure minerals and magnesite ore. The results show that KD-1 can significantly improve the separation effect during reverse flotation. When the dosage of KD-1 is 1500 g/t, the amount of collector can be saved, and the concentrate yield increases obviously. Mechanistic studies have shown that uniform foam, fluidity and surface area are increased by KD-1, and quartz and magnesite can be better separated. Cheng Long^11^ used Materials Studio software to study the strength of different inhibitors on the surface of magnesite. The simulation results were verified by pure mineral flotation tests. The results show that the inhibitory ability of several inhibitors on magnesite is as follows: EDTA > citric acid, tannic acid > water glass > tartaric acid. Feng Qigui et al.^12^ invented a new flotation process using #2 oil, water glass, and oleic acid to obtain a better separation effect by adjusting the dosage of this combination and combining it with better process conditions. Zhou Wenbo^13^ studied the influence of modifiers on the separation of cryptocrystalline magnesite and dolomite. The results show that sodium hexametaphosphate can better inhibit dolomite and realize the separation of Ca and Mg, followed by the inhibitory effect of water glass and sodium fluorosilicate. Sun Tichang^14^ studied the effects of SE as a collector and compared the effects of the alkaline regulator JT and acid regulator ST on reverse flotation for the main gangue mineral quartz in a certain magnesite. This showed that the flotation index is better under alkaline conditions than under acidic conditions. When a large amount of acid regulator is used, it is easy to corrode the equipment. Therefore, an alkaline reverse flotation process is recommended.

Taking magnesite, dolomite, and quartz single minerals as the research object, under the cationic collector ether amine system, the effect of sodium hexametaphosphate (SH), sodium silicate (SS), trisodium phosphate (TP), tannic acid (TN), and monoammonium oxalate (OA) on the floatability of magnesite, dolomite and quartz is studied. Then, the flotation and purification effect at the Haicheng Magnesite Ore Refractory General Plant was studied.

## Test materials and methods

### Test materials

#### Test sample

The magnesite ore used in the test was taken from the Haicheng Pailou mining area (Haicheng Magnesium Ore Refractory Material Plant). The overall colour of the ore is white, and the crystalline structure is dominant. The ore was crushed, screened and mixed in the laboratory to prepare a − 2 mm sample, a small amount of ore sample was taken for laboratory analysis, and the rest was stored for later use.

The single minerals magnesite, dolomite, and quartz were taken from the Haicheng Magnesium Mine Refractory Material Plant, Rongcheng Town Yuxi Ma Steel Dolomite Mine and Inner Mongolia Chifeng Quartz Mine, respectively. Relatively pure lump ore was selected and put through a series of processes such as manual selection, crushing, screening, and grinding. A − 0.106 mm + 0.044 mm particle size was finally obtained for use. Quartz must be soaked in dilute hydrochloric acid and repeatedly washed with distilled water to stabilize the pH value of the solution at approximately 7, and is ready for later use after drying.

**Figure 1:**
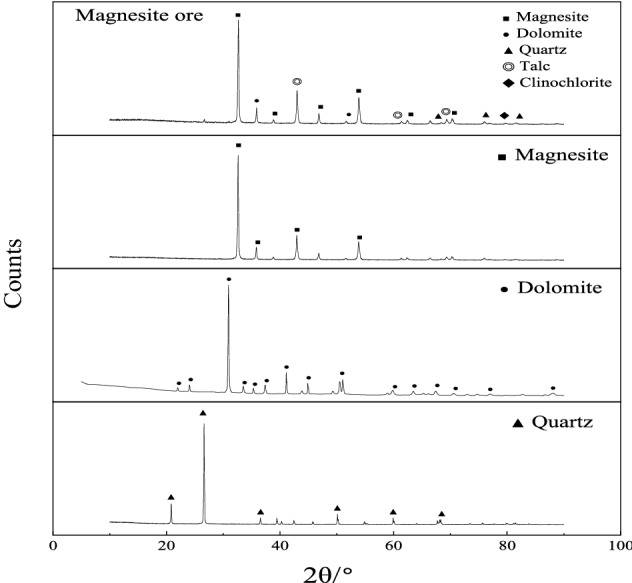
XRD pattern of sample.

The chemical element analyses of magnesite ore, magnesite, dolomite, and quartz single minerals are shown in Table 1, and the X-ray diffraction test results are shown in Fig. 1.

**Table 1:** Chemical composition of magnesite ore, and single mineral magnesite, dolomite, and quartz (wt%).

Sample	MgO	CaO	SiO_2_	Al_2_O_3_	Fe_2_O_3_	LOI	MgO (IL = 0)
Magnesite ore	46.01	0.75	2.38	0.13	0.23	50.5	92.95
Magnesite	47.35	0.25	0.19	0.34	0.17	51.70	98.03
Dolomite	22.24	29.99	0.64	0.34	0.15	46.5	
Quartz	0.08	0.01	99.4	0.22	0.01	–

Table 1 and Fig. 1 show that the MgO content in the magnesite ore is 46.01%, the MgO content is 92.95% (IL = 0), and the impurity SiO_2_ content is relatively high, followed by CaO and Fe_2_O_3_. The pure mineral sample of magnesite contains fewer impurities, in which the MgO content is 47.35%, the MgO content is 98.03% (IL = 0), and the MgCO_3_ content is over 97%. The main component of dolomite minerals is dolomite, and the contents of elemental impurities such as iron, silicon and aluminium are relatively small. The main component of pure quartz minerals is SiO_2_, with a content of 99.4%. Three kinds of minerals meet the requirements of the pure mineral flotation test.

#### Test agent

The reagents used in the flotation test are ether amine, sodium silicate (Na_2_SiO_3_·mH_2_O), and #2 oil (C_10_H_17_O), which are industrial products taken from the manufacturer. Sodium hexametaphosphate, trisodium phosphate, tannic acid and ammonium oxalate are chemically pure or analytically pure and purchased from a reagent sales company.

The ether amine was acidified with hydrochloric acid, and the ether amine hydrochloride solution was used as the flotation collector. The molar ratio of ether amine to hydrochloric acid is 1/1.1, and the amount of acidified ether amine (EAH) collector is based on the amount of ether amine.

### Test method

#### Single mineral flotation test

The pure mineral flotation test uses the hanging tank XFGC type flotation machine used in the laboratory, the flotation tank volume is 30 mL, the flotation machine speed is 1800 r/min, and the test water is distilled water. Each experiment uses 3 g pure minerals and 25 mL distilled water. After adding the regulator and the collector, each mixture is stirred for 1–3 min. After 4 min of flotation, a circulating water vacuum pump was used to filter, dry, and weigh the foam product and the product in the tank.

#### Ore flotation test

The actual ore flotation test uses an XFD flotation machine with a flotation cell volume of 1 L. The flotation machine speed is 1800 r/min, 400 g ore samples are taken each time, and the slurry concentration is 33.33%. The flotation test is carried out with three sequential reverse flotation processes. Chemicals were added in the order of pH adjuster, adjuster, and collector and stirred for 1–3 min. After flotation, the concentrate and tailings were filtered and dried to calculate the product weight and recovery, and then laboratory analysis was conducted.

#### Test method for electrokinetic potential measurement

The electrokinetic potential was measured using a JS94H microelectrophoresis instrument to compare the surface potentials of magnesite, dolomite and quartz in distilled water, the regulator solution, and the collector solution. The ore sample to be tested was ground to − 5 μm with a ZXM-1 vibration mill. A sample of 0.5 g of the mineral was taken. The reagents and distilled water were added to make a solution and stirred with a magnetic stirrer for 5 min. The pH value of the solution was adjusted (HCl or NaOH) and stirred for 5 min before measurement. A total of 0.5 mL of liquid was taken each time and placed into the Zetaprobe measuring cup to measure the electrokinetic potential. This measurement was conducted 4–6 times for each sample, the interference numbers were removed, and the average of the remaining values was taken.

#### Fourier transform infrared (FTIR) spectra

The pure minerals to be tested were added to the aqueous solution containing a certain dose of flotation agent in a certain proportion, the pH was adjusted with hydrochloric acid, and the mixture was fully stirred. It was then filtered, washed with distilled water, and placed in an oven for drying. The prepared sample to be tested was mixed with KBr in an appropriate ratio, pressed into tablets, and then placed in a Nicolet 380 FT-IR infrared spectrometer for measurement.

#### X-ray diffraction measurement

The sample to be tested was ground to − 45 μm, spread onto a flat surface on a slide, and placed in an X-ray diffractometer at room temperature for testing. The diffraction pattern analysis is based on the PDF2-2004 card version of the International Diffraction Data Center (JCPDS-ICDD) of the Joint Committee on Powder Diffraction Data Standards.

## Test results and analysis

### Flotation test of a single mineral under an ether amine system

Under the ether amine system, the flotation behaviour of magnesite, dolomite, and quartz single minerals was studied. The effects of EAH dosage, pulp pH value, and dosage of the regulators SH, SS, TP, TN, OA on the floatability of magnesite, dolomite and quartz were investigated^15^.

#### The influence of EAH dosage on mineral flotation

**Figure 2:**
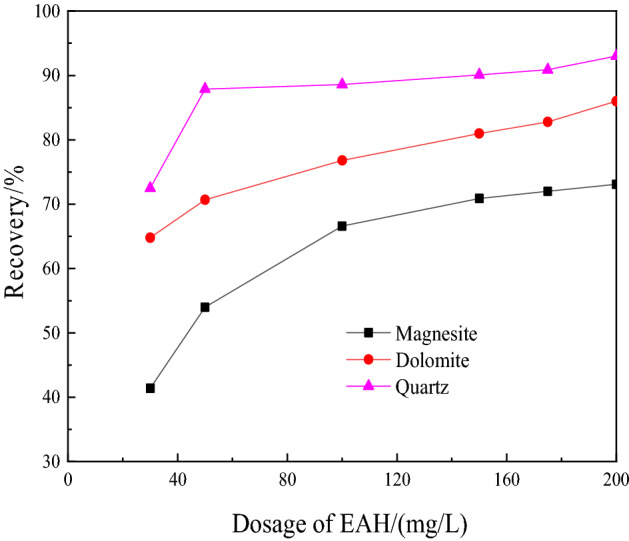
Experimental results of the dosage of the collector.

Under natural pH conditions, when EAH was used as a collector, the effects of different dosages on the floatability of magnesite, dolomite and quartz were investigated. Figure 2 shows the test results.

Figure 2 shows that as the amount of EAH increases, the recovery of the three minerals gradually increases, and quartz has the best floatability. When the amount of EAH is greater than 50 mg/L, the floatation rate is maintained at approximately 90%, the floatability of dolomite is better, the floatability of magnesite is the worst, and the floatation rate is also the lowest. Overall, the amount of the collector ether amine was selected to be 150 mg/L.

#### The influence of pulp pH on mineral floatability

The role of the pH adjuster was to adjust the acid–base environment of the pulp, controlling the surface characteristics of the mineral, and changing the chemical composition of the pulp. H^+^ and OH^−^ ions can change the hydration intensity of the mineral surface by interacting with the mineral surface and adsorb collector ions^16^. Therefore, the pH value of the pulp was an important condition for flotation.

**Figure 3:**
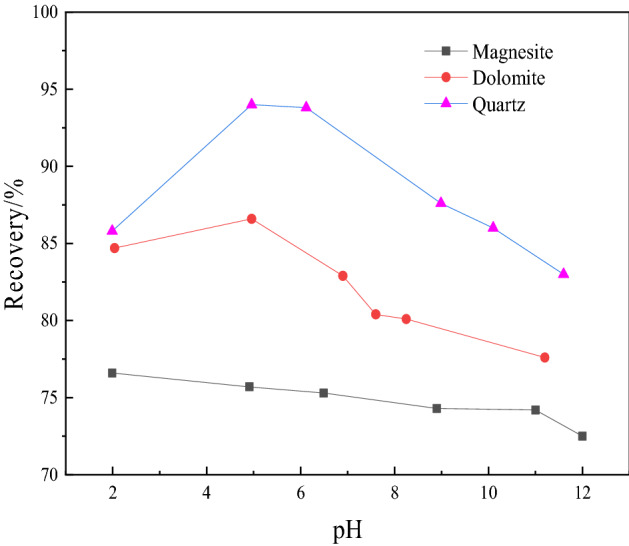
Experimental results of pH.

Under the condition of 150 mg/L collector EAH, the pH value of the slurry was adjusted with HCl and NaOH to investigate the influence of the pH value on the floatability of magnesite, dolomite and quartz. Figure 3 shows the experimental results of pH.

Figure 3 shows that the floatation rate of quartz increases first and then decreases with increasing pH. The floatation rate is the highest when the pH value is 5–6, and it is stable between 92 and 93%. When the pH is 2–5, the floatation rate of dolomite shows an increasing trend. At pH values greater than 5, the floatation rate gradually decreases, but the fluctuation of the floatation rate is small. The floatation rate of magnesite gradually decreases with increasing pH value, but it always remains between 70 and 75%, and the change is small. The results show that in an acidic environment, EAH has the strongest ability to collect quartz, followed by dolomite. Considered comprehensively, the pH should be approximately 5, which is beneficial to the separation of quartz and dolomite from magnesite ore by reverse flotation. At this time, the floatation rates of magnesite, dolomite and quartz are approximately 75.5%, 86.5% and 94%, respectively.

#### The influence of the type and amount of adjuster on mineral floatability

**Figure 4:**
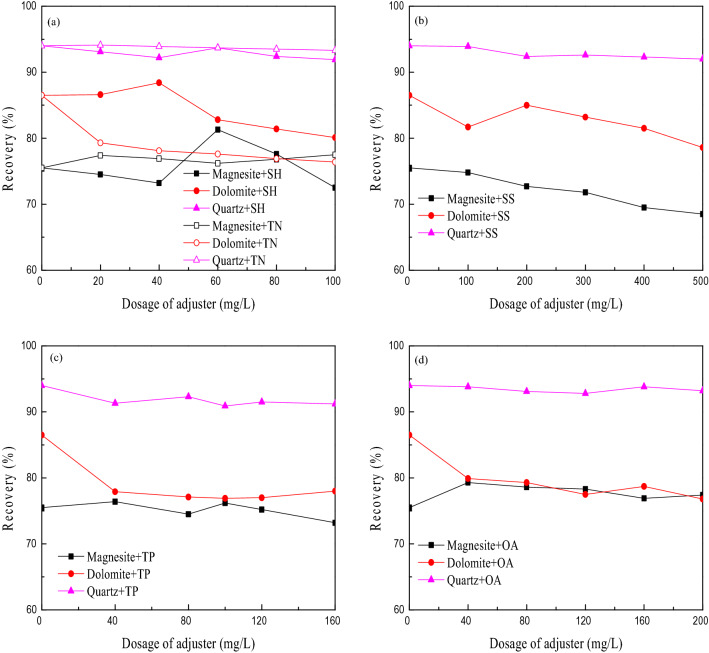
The effect on floatability of the regulators SH and TN (**a**), SS (**b**), TP (**c**), and OA (**d**).

The Na_4_P_6_O_18_^2−^ ions ionized by SH in the slurry have strong activity and easily form stable hydrophilic complexes with Ca^2+^, Fe^3+^ and Mg^2+^ ions, thereby affecting the floatability of minerals containing these ions^17^. SS is often used as an inhibitor of carbonate minerals such as calcite and limestone and gangue minerals such as quartz and silicate. TP, TN, and OA are also commonly used regulators for magnesite flotation, which can effectively inhibit gangue minerals such as quartz and silicate^18^. At the same time, SH and SS also specifically disperse sludge^17^. Under the conditions of a collector dosage of 150 mg/L and a pH of approximately 5, the effects of the dosage of the five regulators on the floatability of magnesite, dolomite and quartz were investigated. Figure 4 shows the test results.

Figure 4a shows that SH has little effect on the recovery of quartz and has a greater impact on the recovery of magnesite and dolomite. When the dosage of SH is less than 40 mg/L, it can activate dolomite. When the dosage is 40 mg/L, the activation effect on dolomite is most obvious. When the dosage of SH is greater than 40 mg/L, it can inhibit dolomite. SH has an overall inhibitory effect on magnesite, and only when the amount of SH is approximately 60 mg/L can magnesite be activated.

When TN is added, the floatation rate of quartz increases slightly, but the increase is small. TN has an activating effect on magnesite and an inhibitory effect on dolomite.

Figure 4b shows that SS has a certain inhibitory effect on magnesite, dolomite and quartz. As the amount of SS increases, the recovery of quartz changes little, and the recovery of dolomite first decreases, then increases and then decreases again. As the dosage of SS increased from 100 to 500 mg/L, the floatation rate of magnesite gradually decreased from 76.32 to 68.21%. SS has an inhibitory effect on magnesite, and its strength is greater than that of quartz.

Figure 4c shows that TP has little effect on the recovery of magnesite and quartz but has a great effect on dolomite, and TP has an inhibitory effect on dolomite.

Figure 4d shows that with increasing OA dosage, the recoveries of magnesite, dolomite and quartz all decrease slightly. It has little effect on the recovery of quartz and magnesite and has a greater impact on the recovery of dolomite.

Considering Fig. 4a,d, of the five kinds of regulators, TN activates quartz; SH has little effect on the floatability of quartz; TP, SS and OA inhibit quartz; the order of inhibition effect is: TP > OA > SS. The five regulators have little effect on the floatability of magnesite. SH has an inhibitory effect on magnesite. Only when the dosage of SH is approximately 60 mg/L can magnesite be activated. TN has an activating effect on magnesite; TP has little effect on the floatability of magnesite; SS and OA have an inhibitory effect on magnesite; and the five modifiers have little effect on the floatability of magnesite. When the dosage of SH is less than 40 mg/L, it can activate dolomite, and when the dosage of SH is greater than 40 mg/L, it can inhibit dolomite. TN, TP, OA and SS have inhibitory effects on dolomite, and TN, TP and OA have equivalent inhibitory effects on dolomite and are stronger than SS.

The lower the content of SiO_2_ in the magnesite concentrate is, the higher the quality of the concentrate. A lower CaO content in the concentrate is preferred to maintain the CaO content over two times the SiO_2_ content. Under the condition of CaO/SiO_2_ ≥ 2, the quality of the concentrate with a low CaO content is better.

Depending on the content of silicon and calcium in magnesite flotation, the process and technology used are different. For ores with low SiO_2_ content (generally less than 2–3%) and low CaO content (generally less than 0.5%). The cationic reverse flotation process of inhibiting magnesium and floating silicon is adopted, and the floating calcium must be inhibited. The purpose of flotation is only to remove silicon, and calcium enters the concentrate. For ores with low SiO_2_ content (generally less than 2–3%) and CaO content 0.6–1%, the cation reverse flotation of magnesium inhibiting floatation of silicon is adopted, but calcium needs to be activated. While silicon is removed, part of the calcium is also removed. For high-silicon and high-calcium ore, cationic reverse flotation is used to remove most of the silicon, and then anionic positive flotation is used to remove calcium and a little silicon that is difficult to remove in the reverse flotation stage.

### Flotation test of a certain magnesite ore in the Haicheng area

The SiO_2_ content in the magnesite ore is 2.38%, and the CaO content is 0.75%. Therefore, it is more appropriate to use a single reverse flotation process to float silicon, and to inhibit magnesium and activate calcium to remove silicon impurities while removing some calcium.

**Figure 5:**
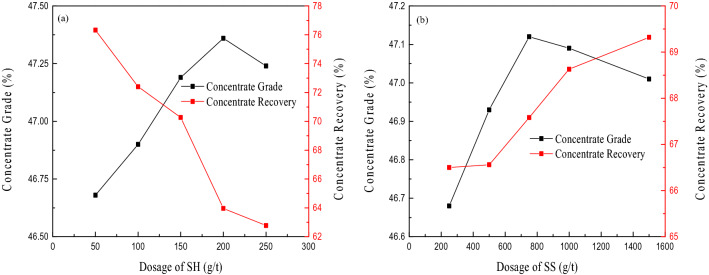
Influence on flotation effect of SH (**a**) and SS (**b**).

The pure mineral test shows that a single reverse flotation process with SH and SS as regulators can achieve effective removal of silicon and part of the calcium at the same time. After the condition test, it was determined that the suitable grinding fineness was − 0.074 mm (accounting for 85%), the pulp pH was 5, and the flotation process consisted of three reverse flotation processes. The amount of collector EAH was 150 g/t (75 g/t, 50 g/t, 25 g/t for 3 flotation cycles). Under these conditions, the influence of SH and SS on the flotation effect was investigated, and Fig. 5 shows the test results.

Figure 5a shows that as the amount of SH increases, the MgO grade first increases and then decreases, and the recovery continues to decrease. Considering the concentrate grade and recovery, the SH dosage was determined to be 150 g/t. At this time, the concentrate grade is 47.19%, and the concentrate recovery is 70.3%. Figure 5b shows that when the amount of SS is increased from 250 to 750 g/t, the recovery of the MgO grade shows an increasing trend. As the amount of SS continues to increase, the recovery of concentrate continues to increase, but the grade of concentrate MgO begins to decrease. When the amount of SS was 1500 g/t, the concentrate grade was 47.01%, and the concentrate recovery was 69.3%. Comparing SH and SS regulators, under the same condition of obtaining concentrate grade, the recovery when using SH as regulator is 1% higher than when using SS as regulator.

### Research on the role of agents and minerals

#### Electrokinetic potential analysis

In aqueous solution, minerals dissolve selectively so that the surface of the minerals has a certain amount of positive and negative charges. The adsorption of the agent and the minerals will be affected by the electrical properties and potential of the mineral surface. In addition, the degree of dispersion of mineral particles in the slurry is also controlled by the electrical properties of the mineral surface, and the degree of dispersion of mineral particles will also affect the flotation effect^19^.

**Figure 6:**
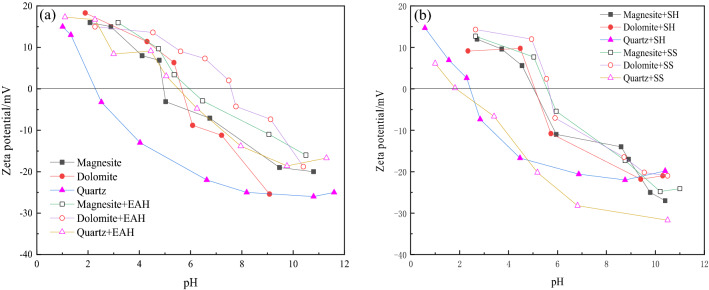
Zeta potential with pH change.

The pH was adjusted with HCl and NaOH. The electrokinetic potential values of three kinds of pure minerals, minerals and EAH (120 mg/L), minerals and SH (20 mg/L), and minerals and SS (200 mg/L) were measured. Figure 6 shows the zeta potential as the pH changes.

Figure 6a shows that the zero electric points of magnesite and dolomite are approximately pH 5.1 and 6.0, respectively, and the isoelectric point of quartz is pH = 2.1. In pH ranges where pH > 2.1, the surface of quartz is negatively charged, so it is more suitable to use a cation collector to collect quartz. In an acidic environment, magnesite and dolomite easily dissolve, so the zeta potentials of magnesite and dolomite in strong acids may be affected. When EAH is added, the separated OH^−^ is increased, ether amine is adsorbed on the surface of magnesite, and OH^−^ will generate Mg(OH)_2_ on the surface of a small amount of magnesite, which will increase the electrokinetic potential of the magnesite surface. The zeta potential of dolomite decreases with increasing alkalinity as a whole. After adding ether amine, the zero electric point of dolomite moves in the alkaline direction. When the dosage of EAH is 120 mg/L, the zero electric point of dolomite is pH = 7.6. The addition of EAH causes the zero electric point of magnesite, dolomite, and quartz to move obviously in the alkaline direction. The movement range is quartz > dolomite > magnesite, indicating that the adsorption capacity of the collector on the surface of the three minerals is quartz > dolomite > magnesite. This result is consistent with the results of the flotation test (Fig. 2).

Figure 6b shows that the addition of SH significantly reduces the zeta potential of magnesite, and the zero electric point shifts slightly toward alkaline. The isoelectric point of dolomite moves in the acidic direction with the addition of SH, and the pH at the zero electric point has a slight change. The zeta potential of quartz is hardly affected by the addition of SH. The zeta potential of magnesite was only slightly reduced by SS, and the isoelectric point pH was approximately 5.8. The addition of SS did not affect the electrical properties of the dolomite surface, and the zero electrical point position did not change. When SS is added, the zeta potential of quartz drops significantly.

SS mainly exists in the form of Si(OH)_3_ at pH = 5–6, followed by a small amount of SiO(OH)^3−^, and mostly exists in SiO_2_(OH)_2_^2−^ in alkaline environments. It is not difficult to explain that in the potential test, water glass has a significant effect on reducing the zeta potential of the mineral surface under alkaline conditions. This is mainly caused by the adsorption of SiO(OH)^3−^ and SiO_2_(OH)_2_^2−^ on the surface of minerals by physical electrostatic forces.

#### Infrared spectrum analysis

**Figure 7:**
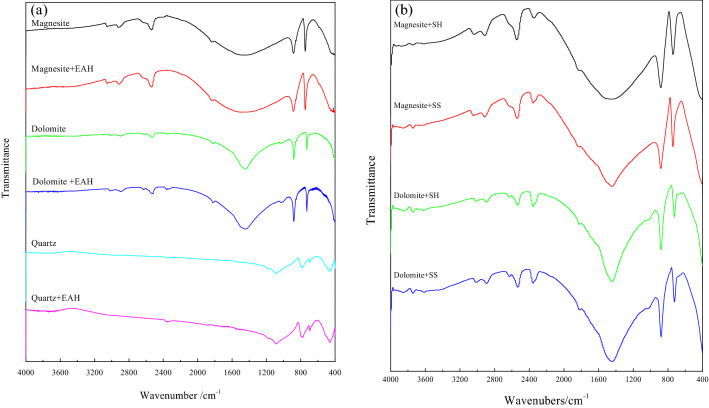
Infrared spectra of minerals and EAH (**a**), SH, SS (**b**).

Figure 7 shows the results of infrared spectroscopy analysis before and after the interaction of three pure minerals with EAH, and magnesite and dolomite with SH and SS.

Figure 7a shows magnesite, dolomite and quartz before and after the interaction with EAH. The peak positions of quartz, dolomite, and magnesite are not shifted, indicating that physical adsorption occurs between the three minerals and EAH, and there is no chemical adsorption, which is consistent with the previous zeta potential analysis results. This may be due to the electrostatic adsorption of RNH_3_^+^ and (RNH_3_)_2_^2+^ plasma on the surface of minerals in the amine chemical solution.

Figure 7b shows that after the interaction of magnesite and SH, a tiny new peak appeared at 710 cm^−1^, and the peak intensity at 2538 cm^−1^ increased^20^. After the action of dolomite with SH, new peaks appeared at 682 cm^−1^ and 1103 cm^−1^, of which 1103 cm^−1^ was the P–O stretching vibration peak. The peak intensity at 2531 cm^−1^ also increased significantly, and the P=O stretching vibration peak at 1277 cm^−1^ was not very obvious because it was near the antisymmetric stretching vibration frequency of CO_3_^2−^ 1453.79 cm^−1^^21^. Therefore, SH is chemically adsorbed with magnesite and dolomite, but the adsorption effect with dolomite is obviously stronger than that of magnesite, which changes the floatability of minerals to varying degrees.

After the action of SS, the peak of magnesite at 2536.89 cm^−1^ changed and shifted significantly, and the characteristic peak of CO_3_^2−^ also changed significantly. After the interaction of dolomite with SS, two Si–OH and Si–O–Si stretching vibration peaks appeared at 3449 cm^−1^ and 683 cm^−1^. The peak shape of the absorption peak (1440 cm^−1^) of the CO_3_^2−^ group of dolomite itself also became sharp. The appearance and changes of the abovementioned various new peaks indicate that SS and the two minerals are chemically adsorbed, and the adsorption effect of water glass on dolomite is obviously better than that on magnesite.

#### Charge analysis and solution chemistry of mineral surfaces

Magnesite and dolomite carbonate ion crystals and carbonate minerals dissolve in acidic water, and the dissolution rate is negatively related to the pH value of the slurry. The pH value of the slurry is affected by the concentration of CO_2_ and mineral lattice ions in the matrix. The order of ion concentration of magnesite in pH 5 aqueous solution is Mg^2+^ > MgOH^+^  > H_2_CO_3_ > HCO_3_^−^, and the order of ion concentration of dolomite in pH 5–6 aqueous solution is Ca^2+^ > Mg^2+^ > MgOH^+^  > CaOH^+^ > HCO_3_^−^ > H_2_CO_3_ > CO_2_^2−^.

The main component of SH is (NaPO_3_)_6_, which has strong adsorption^22^. SH is dissolved in water, and the research results show that when the pH is less than 2.15, H_3_PO_4_ is the dominant component. H_2_PO_4_^−^ is the dominant component in the pH range of 2.15 < pH < 7.2. The pH range in which HPO_4_^2−^ is the dominant component is 7.2 < pH < 12.35, and PO_4_^3−^ dominates when pH > 12.35. Therefore, when the flotation test is between pH = 5–6, the phosphate anion and a small amount of hydrolysed product H_2_PO_4_^−^ dominate in the SH solution.

SS is easy to dissolve in water. Some research results show that when the pH is less than 9.4, the dominant component is Si(OH)_4_; when pH ≥ 9.4, SiO(OH)_3_^−^ is dominant; and when pH ≥ 12.6, SiO_2_(OH)_2_^2−^ is dominant. Therefore, when the flotation test is between pH = 5–6, it can be considered that the monosilicic acid Si(OH)_4_ in the water glass is an effective component with an inhibitory effect^23^.

The chemical equilibrium of ether amine in aqueous solution is:

$${\text{RNH}}_{{2}} \left( {\text{s}} \right) \leftrightarrow {\text{RNH}}_{{2}} \left( {{\text{aq}}} \right),$$

$${\text{RNH}}^{{{3} + }} \leftrightarrow {\text{RNH}}_{{2}} ,$$

$${\text{2RNH}}_{{3}}^{ + } \leftrightarrow ({\text{RNH}}_{{3}} )_{{2}}^{{{2} + }} ,$$

$${\text{NH}}_{{3}}^{ + } + {\text{RNH}}_{{2}} \leftrightarrow \, ({\text{RNH}}_{{2}} \cdot {\text{RNH}}_{{3}} )^{ + } .$$

The research results show that in an acidic pH environment, amines mainly exist in the form of ions and when pH > 5, amine molecules gradually form. The existence of amine molecules provides the possibility for the formation of "acid soap" dimers. That is, the greater the concentration of amine molecules in the flotation slurry with a pH of approximately 5, the main forms of amines are RNH_3_^+^ and (RNH_3_)_2_^2+^, as well as a small amount of RNH_2_ in molecular form.

## Conclusions

1. In the collector ether amine system, at a slurry pH of 5.0, ether amine has a certain collection effect on magnesite, dolomite, and quartz, and the collection capacity gradually increases with the increase in the amount of ether amine. Quartz has the highest floating rate among the three pure minerals, followed by dolomite and magnesite. The regulators sodium hexametaphosphate, sodium silicate, trisodium phosphate, tannic acid and ammonium oxalate have a certain effect on the floating rate of the three minerals. For reverse flotation to remove gangue mainly containing silica gangue minerals, sodium hexametaphosphate is the most effective, followed by sodium silicate.
2. The actual ore flotation adopts a single reverse flotation process. At a grinding fineness of − 0.074 mm, the content was 85%, the reverse flotation pH was 5.0, and the ether amine dosage was 150 g/t (75 g/t for the first stage, 50 g/t for the second stage, 25 g/t for the third stage). The amount of hexametaphosphoric acid used alone was 150 g/t, and the process of one coarse and two fine flotations was adopted. The MgO content of the concentrate is 47.19%, the SiO_2_ grade is less than 0.25%, the CaO grade is approximately 0.6%, and the concentrate yield is greater than 70%.
3. The surface electrical property measurements, infrared spectroscopy analyses, and chemical solution calculations show that there are both chemical adsorption and physical adsorption on the surface of sodium hexametaphosphate and water glass and minerals (dolomite and magnesite). Acidified ether amine, magnesite and dolomite are mainly physically adsorbed. When pH = 5.0–6.0, the phosphate anion and a small amount of hydrolysis product H_2_PO_4_^−^ in the sodium hexametaphosphate solution are dominant. Monosilicic acid Si(OH)_4_ in the water glass solution is the main component of water glass in flotation.
